# Anthropogenic Neighborhood Impact on Bacterial and Fungal Communities in Polar Bear Feces

**DOI:** 10.3390/ani13132067

**Published:** 2023-06-22

**Authors:** Maksim V. Vecherskii, Tatiana A. Kuznetsova, David R. Khayrullin, Aleksandr A. Stepankov, Svetlana M. Artemieva, Pavel V. Chukmasov, Evgeny A. Ivanov, Ivan A. Mizin, Ilya N. Mordvintsev, Nikita G. Platonov, Aleksandr A. Pashali, Artem I. Isachenko, Renata E. Lazareva, Ksenia M. Shestakova, Viatcheslav V. Rozhnov

**Affiliations:** 1 A.N. Severtsov Institute of Ecology and Evolution, Russian Academy of Sciences, Moscow 119071, Russia; 2 Russian Arctic National Park, Arkhangelsk 163051, Russia; 3 PJSC Rosneft Oil Company, Moscow 117997, Russia; 4 LLC Arctic Research Center, Moscow 119333, Russia; 5 Institute of Translational Medicine and Biotechnology, I.M. Sechenov First Moscow Medical University, Moscow 119435, Russia

**Keywords:** polar bear, intestinal microbiome, NGS

## Abstract

**Simple Summary:**

Polar bears are native inhabitants of the Arctic ice. An increase in the ice-free season forces polar bears ashore and prompts them to invade human settlements and feed on human waste. Since their usual food objects (seals)differ extremely from human waste, the enforced diet shifts are considered to be stressful and may lead to intestinal dysbiosis and digestive disorders. To assess the resulting changes, we compared the gut bacterial community of bears feeding on their natural food and bears visiting the human waste dump. To our knowledge this is the first investigation of the fugal community in polar bears’ intestines. It turned out that feeding at the dump does not facilitate the development of dysbiosis in polar bears, but, on the contrary, leads to the formation of an adaptive microbial community similar to that of omnivorous animals. This is also confirmed by the high fat reserves in the animals attending the dump, which increases their chances of surviving the starvation period. However, in the structure of the fungal community, facultative pathogenic yeast species appear, which are theoretically capable of provoking various infections. Moreover, polar bears that do not visit human settlements do not harbor a specific fungal community.

**Abstract:**

Climate changes cause a dramatical increase in the ice-free season in the Arctic, forcing polar bears ashore, closer to human settlements associated with new and non-natural food objects. Such a diet may crucially transform the intestinal microbiome and metabolism of polar bears. The aim of this study was to characterize changes in the gut bacterial and fungal communities resulting from the transition to anthropogenic food objects by the means of 16S and ITS metabarcoding. Thus, rectal samples from 16 wild polar bears from the Kara–Barents subpopulation were studied. Human waste consuming resulted in a significant increase in the relative abundance of fermentative bacteria (Lactobacillaceae, Leuconostocaceae, and Streptococcaceae) and a decrease in proteolytic Enterobacteriaceae. However, the alpha-diversity parameters remained similar. Also, for the first time, the composition of the fungal community of the polar bear intestine was determined. Diet change is associated with the displacement of eurybiontic fungi (*Thelebolus*, *Dipodascus*, *Candida* (*sake*), and *Geotrichum*) by opportunistic *Candida (tropicalis)*, *Kazachstania*, and *Trichosporon*. Feeding on human waste does not cause any signs of dysbiosis and probably leads to adaptive changes in the bacterial microbiome. However, the emergence of fungal facultative pathogens increases the risk of infections.

## 1. Introduction

The polar bear (*Ursus maritimus*), as the apex predator, occupies the top of the Arctic food chain, and can thus be used as an indicator of the state of the Arctic ecosystems. The main habitat of this species is the Arctic Sea ice [1], which also houses the main preys of the polar bear, the ringed seal (*Pusa hispida*) and the bearded seal (*Erignathus barbatus*) [1]. However, nowadays, the climate change that is mostly pronounced in the Arctic results in a catastrophic loss of the natural habitats for the polar bear. A reduction in the drifting ice area in the summer and the duration of the presence of one-year ice are recognized as the main threats to the well-being of polar bear populations [2,3,4,5]. The Barents Sea is one of the regions with the most rapid changes in ice conditions that hardly give polar bears the opportunity to adapt to the changing environments [6,7]. Therefore, the climate change and the subsequent earlier ice melting in the spring provoke the bears to stay on shore [8] during unfavorable conditions, where they are unable to feed on their natural prey. The absence of natural food objects forces bears to feed on resources available on the shore, such as coastal kelp aggregations, herbaceous plants, eggs and the chicks of seabirds, small rodents, remains of marine mammals washed ashore, reindeers, etc. Such a diet can lead to the exhaustion of bears and even starvation [8,9,10,11,12,13,14]. Exhausted male bears may even prey on their conspecifics [15]. Moreover, the invasions of human settlements by bears, their contacts with domestic animals, feeding on human supplies and waste dumps have become more frequent. On the one hand, such behavior saves the bears from starvation; on the other hand, it increases the risks of digestive and metabolic disorders, as well as microbial and viral infections.

It is well known that the diet crucially affects the composition of the intestinal microbiome that in turn influences the immune system, digestion, and the whole metabolic ability of the animal. It was previously shown that the transition of bears from the natural so-called ice diet to terrestrial food objects leads to significant changes in the composition of their intestinal microbiome [16]. However, despite the significant intrapopulation variability in the composition of intestinal microorganisms of the polar bear, the core microbiome of the intestinal community can be distinguished. This includes representatives of *Escherichia*/*Shigella*, *Corynebacterium*, *Clostridium*, *Collinsella*, and Lachnospiracea species, and some others [16]. On the ice diet, the intestinal community of the polar bear is dominated by the representatives of the genera *Clostridium* and *Bacteroides*, while the shift to the terrestrial diet changes the intestinal dominants: the role of representatives of the families Erysipelotrichaceae, Lachnospiraceae, and Peptostreptococcaceae increases. Moreover, these changes are considered to be adaptive in their nature [17]. It is assumed that the encounter with novel microorganisms (and possibly, pathogens) increases the immune system activity and may affect the physiological state of the animal [18].

Also, the role of fungi in the microbiome structure of the gastrointestinal tract is considered underestimated [19]. These microscopic eukaryotes specifically affect immune system activation that is impossible for all the known prokaryotes. First, this includes the induction of T helper 17 cells, which are crucial for the pathogenesis of many autoimmune diseases [20,21]. Since the terrestrial diet and, especially, human waste contains more carbohydrates than the ice diet, the increased role of fermenting fungi (such as representatives of the class Saccharomycetes) in the bear’s intestine is very likely. The transmission of pathogenic yeasts from domestic animals and pets is also possible.

The aim of this work was to identify changes in the gut microbial community in wild polar bears of the Kara–Barents subpopulation, resulting from the dietary shift to human food waste objects.

We predict that such dietary shifts will drive significant changes in the intestinal microbiome, which result in either dysbiosis or specific adaptive changes of the community. In addition, we planned to expand the current understanding of the normal polar bear gut microbiome with previously unpublished data on the Kara–Barents subpopulation. Additionally, to our knowledge, this is the first description of the fungal community composition of the polar bear intestine.

We also expect that feeding on human waste may reduce starvation and preserve fat amount during the increasing ice-free period.

## 2. Materials and Methods

### 2.1. Sampling

**Figure 1:**
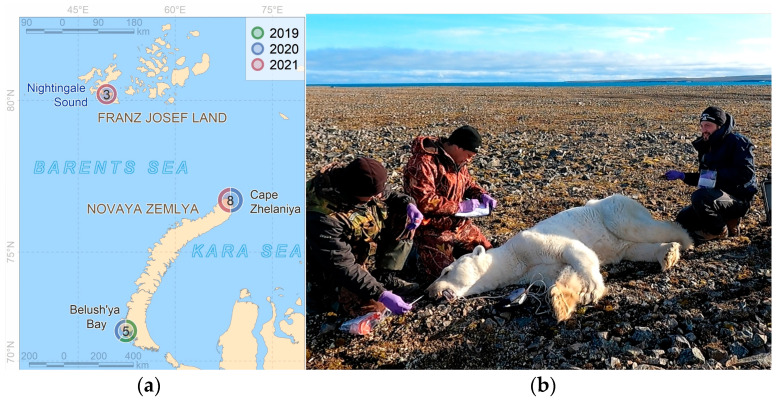
Location of the sampling sites (**a**) and the preparations for the sampling procedure (**b**). Land polygons for the basemap (**a**) are from “Data Derived from OpenStreetMap for Download” (https://osmdata.openstreetmap.de/data/land-polygons.html (accessed on 19 April 2023), updated 2022-03-07T04:39), under the Open Database License “ODbL” by the OpenStreetMap Foundation. Numbers in the circles indicate the number of studied bears.

Our studies of the Kara–Barents subpopulation of polar bears (*Ursus maritimus*) were carried out in 2019–2021 at three model sites with completely different local environments (Figure 1) and, subsequently, animal foraging habits.

1. The Franz Josef Land archipelago (3 individuals). The samples were collected at the beginning of the ice melting period (summer). The bears usually prey on adult seals and cubs in dens in between the islands in the southern part of the archipelago during this period.
2. Novaya Zemlya archipelago, Severny Island, Cape Zhelaniya (8 individuals). The samples were collected during the ice-free period (summer–autumn), during which the bears were fasting and feeding opportunistically on the remains of a walrus washed ashore, grass, and kelp.
3. Novaya Zemlya archipelago, Yuzhny Island, Belushya Bay, in the area of a large village of Belushya Guba (5 individuals). The samples were collected when the Barents Sea coast was free of landfast ice (autumn). The bears were feeding on human food waste landfill.

Due to the limited number of the found polar bears, we recognized and investigated the impact of only two main ‘types’ of the bears’ diet: the natural diet (N-bears) (although we understood the variability related to the different habitat conditions) and the human-affected diet, the food waste landfill (A-bears).

The samples for microbiological studies were collected from polar bears after their immobilization using a DAN-Inject JM-25 rifle. A combination of medetomidine (Meditin, Apicenna, Russia, dose 0.06 mg/kg of animal body weight) with a mixture of tiletamine/zolazepam (Telazol, Zoetis Services LLC, Parsippany, NJ, USA, 2 mg/kg of animal body weight) was used as an immobilizing drug. These veterinary drugs were certified for use on the territory of the Russian Federation and have been successfully applied in our previous studies on large predators since 2008. For each immobilized individual, the time and geographical coordinates of the place of capture were recorded (using GPS receivers); air temperature at the time of capture and a set of additional meteorological and environmental parameters were detected. Biometric measurements were recorded, including sex and body mass as part of long-term population assessments. When we were unable to weight the bears, the mass was calculated based on straight-line body length and axillary girth [22]. The condition of each bear was assessed based on the generally accepted five-point scale (Polar Bear Score Card: A Standardized Fatness Index) [23]. According to this scale, the appearance of the polar bears can be skinny, thin, average, fat, or very fat. The appearance of the polar bear was compared with the images and descriptions on this Score Card, paying particular attention to visible fat deposits on the body, protruding bones of the pelvis and shoulder girdle, etc. The body condition index (BCI) was calculated for each bear from body mass and straight-line body length [24]. The age class of every individual bear was estimated based on size, tooth wear, and dependence on mother’s milk. We distinguished age classes: cub (fully dependent young 0–1 years), juvenile (dependent young 2–3 years), subadult (independent young 2–5 years), and adult (bear of reproductive age ≥6 years) [25]. Excrements (samples) were taken from the rectum of the animal by a hand in a medical glove using a lubricating gel. After completing all the necessary procedures, the animal received an intramuscular injection of atipamezole hydrochloride (Antimedin, Apicenna, Russia, dose 0.24 mg/kg), which leads to a quick and safe recovery from anesthesia.

All manipulations with bears were carried out in accordance with the protocol approved by the ethics commission of the Severtsov Institute of Ecology and Evolution of the Russian Academy of Sciences (no. 37 dated 25 May 2020).

Prior to capture, the polar bears were observed only for several days. After immobilization, the female bears were tracked using satellite telemetry data. Marked with ear marks, the males remained in the area of immobilization and were observed repeatedly in the area of the waste landfill.

All samples were placed in plastic bags and kept in cooling bag. No later than 1 h after sampling, the samples were frozen at −18 °C in a freezer. The samples were transported for their subsequent storage in the cryochambers of the Institute in a frozen form.

### 2.2. Microbiome Studies

For each sample, fecal DNA isolation was performed in duplicate with the NucleoSpin Soil Kit, MACHEREY-NAGEL (Germany), in accordance with the manufacture’s instruction. The V3–V4 region of the 16S rRNA gene was amplified in duplicate for all the studied bears (11 N and 5 A) using the F (341) 5′-CCT ACG GGN GGC WGC AG-3′/R (805) 5′-GAC TAC HVG GGT ATC TAA TCC-3′ primer set. The ITS region of the rRNA gene was amplified using the ITS 1F 5′-GCA TCG ATG AAG AAC GCA GC-3′, ITS1R 5′-TCC TCC GCT TAT TGA TAT GC-3′ primer set for 13 polar bears (8 N and 5A). The libraries were sequenced on Illumina MiSeq 2 × 300 bp sequencing. A total of 1,124,324 reads were obtained after sequencing the amplicon libraries for 16S rRNA gene and 1,270,568 reads for the ITS region. The research was performed using equipment of the core centrum Genomic Technologies, Proteomics, and Cell Biology of the All-Russian Research Institute for Agricultural Microbiology of the Russian Academy of Sciences (Sankt-Petersburg, Russia). Raw sequence data were processed to remove adapters, primers, and primer-free reads using Qiime cutadapt. The DADA2 pipeline [26] was applied to denoise the reads, trim the reads based on quality scores (Q25 ≥ 100%), and merge the paired-end reads. Subsequently, the denoised sequences were checked for chimeric sequences using Qiime-Vsearch; the latter were excluded from the downstream analyses. The remaining sequences were clustered into operational taxonomic units (OTUs) using the open_reference algorithm against the Silva132 (for bacteria) and Unite ver.8 4 February 2020 (for fungi) reference databases based on a 97% consensus threshold. All annotations were manually checked by Blast PubMed. De novo OTUs were also manually assigned by Blast Genbank PubMed. The OTUs with identical annotations at the species level were demultiplicated. Obsolete taxonomic data from Silva132 were checked.

The functional profiles of microbial communities were predicted using PICRUSt version 2.0. [27] with the KEGG (Kyoto Encyclopedia of Genes and Genomes) Orthology database [28]. The OTU table was normalized to correct the number of multiple 16S rRNA gene copies using the Silva 132 reference database. Principal component analysis (PCA) was performed to overview trends in the activity of the microbial communities in bears from N-group and A-group using Python scikit-learn v.1.2.2 package.

Basic statistics (mean ± SD) were calculated. The samples were checked for normal distribution (Shapiro–Wilk test) and a non-parametric Mann–Whitney U test was used to evaluate the differences in the abundance of the gut microbiota, as well as alpha-diversity indexes between the A- and N-groups of polar bears using Statistic 8.0 (Statsoft, Tulsa, OK, USA); the indexes of alpha-diversity, PCoA of beta-diversity, and PERMANOVA were computed using Qiime2.

## 3. Results

### 3.1. State Conditions of the Polar Bears

All the studied bears varied in their sex, age, and weight (Table 1). Moreover, we found statistically significant difference in the subjective fatness index (FI) of bears from the two studied groups: for the animals feeding at the human waste landfill, the FI rating was higher (i.e., better) than in the animals on their natural diet (Mann–Whitney U Test, *Z* = 2.2091, *p* = 0.027). However, the body condition index (BCI), calculated from the mass and size parameters of the bears, did not differ significantly (Mann–Whitney U Test, *Z* = −1.5556, *p* = 0.1988).

### 3.2. Gut Bacterial Community of the Polar Bears

In total, 567 OTUs (355 OTUs for the A-bears (*n* = 5) and 422 for N-bears (*n* = 11)) were received. Nevertheless, the average number of the observed OTUs in the A-bear group microbiome was higher than that of the N-bear group (Table 2); however, this difference was not statistically significant (*p* > 0.05).

Although the mean alpha-diversity indexes did not differ statistically among the studied groups of bears, N-bears were characterized by higher values of Simpson and Shannon indexes (Table 1). Also, the inter-individual variations in the values of these indexes for the A-group were higher than for the N-group (Shannon: 1.23 ± 5.23 and 2.86 ± 4.61; Simpson: 0.37 ± 0.94 and 0.75 ± 0.94 for A and N-groups, respectively).

**Figure 2:**
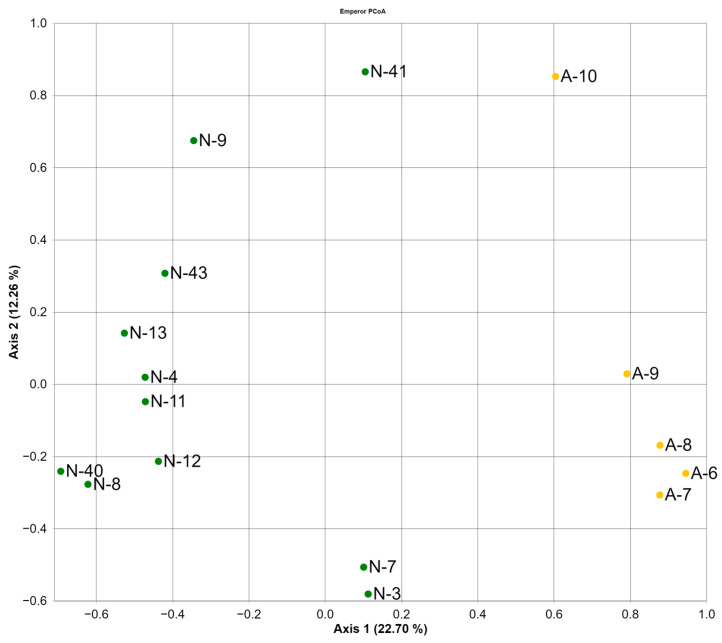
Principal coordinate analysis (PCoA) based on Bray–Curtis dissimilarity between the samples. (Hereinafter: A, group of polar bears that feeds on anthropogenic food waste landfill; N, bears that feed on their natural diet).

The beta-diversity was estimated using the ordination of the microbiome composition in principal coordinate analysis (PCoA) based on Bray–Curtis measurements (Figure 2). The graph shows a clear separation between the A-group and the N-group (PERMANOVA pseudo-F test = 3.5629, *p* = 0.001). The distinction is due to differences in the composition of the microbiome at all taxonomic levels, which will be shown below. There are no patterns related to gender, age, or reproductive condition (PERMANOVA pseudo-F *p* > 0.05).

**Figure 3:**
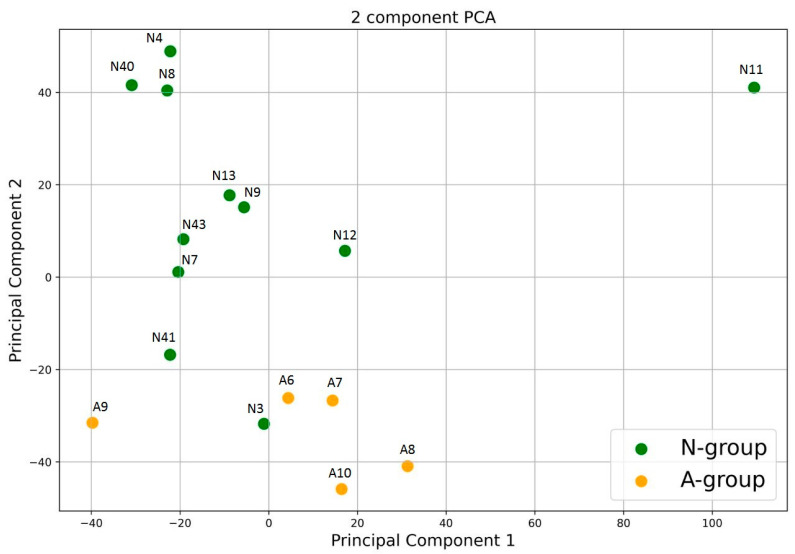
Principal component analysis of metabolic data of the polar bear intestinal communities via KEGG.

As phylogenetically distant microorganisms may be metabolically synonymous, we performed PCA of the predicted functional profiles (Figure 3). It showed some separation in metabolic profiles between the A-group and N-group, although the differences were not as clear as in the phylogenetic analysis. Similar to phylogeny, there are no patterns related to gender, age or reproductive condition.

**Figure 4:**
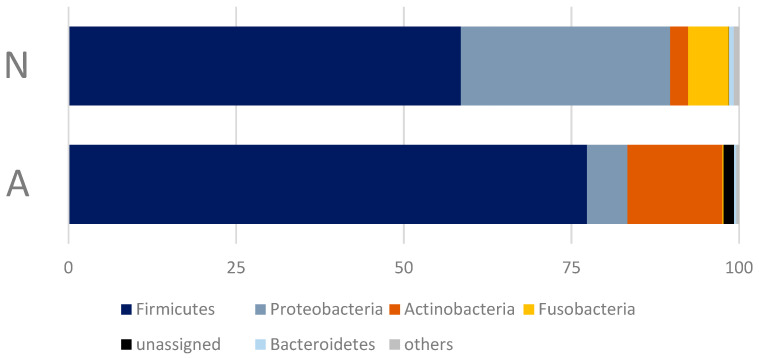
Relative abundance of the five most abundant bacterial phyla, averaged across all samples within each of the studied groups of polar bears.

All annotated OTUs belong to 14 bacterial phyla: Firmicutes, Proteobacteria, Actinobacteria, Fusobacteria, Bacteroidetes, Cyanobacteria, Patescibacteria, Chloroflexi, Saccharibacteria, Epsilonbacteraeota, Planctomycetes, Euryarchaeota, Verrucomicrobia, and Chlamydiae. However, only seven of them were detected in the amount of more than 0.5% (Figure 4), and only four of them were predominant, comprising about 98.5% of the reads (Table S1).

**Figure 5:**
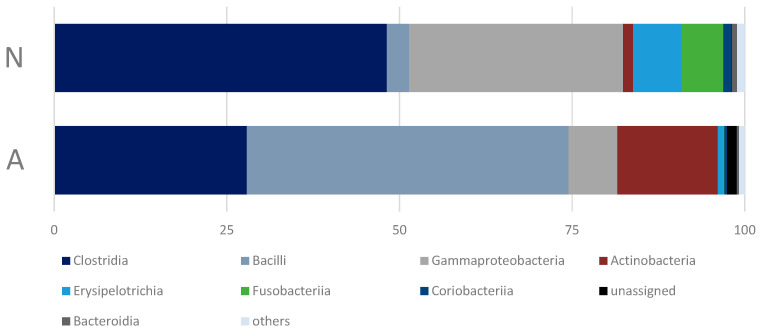
Relative abundance of the eight most abundant bacterial classes (average abundance over 0.5%), averaged across all samples within each of the studied groups of polar bears.

The differences in the bacterial microbiome communities of the bears that consume human waste (A-bears) and those feeding on a natural diet (N-bears) were found to be significant only for the phylum Proteobacteria (Mann–Whitney U Test, *Z* = 2.60563, *p* = 0.009171). In addition, it should be noted, that despite the lack of the statistically significant differences in the proportion of the phylum Firmicutes, its structure in the microbiome of the A-bears changed crucially at the bacterial class level: Clostridia and Erysipelotrichia were replaced by Bacilli (Figure 5). Of the 23 bacterial classes detected in the fecal microbiome of the studied polar bears, eight accounted for 98.57% of the reads.

**Figure 6:**
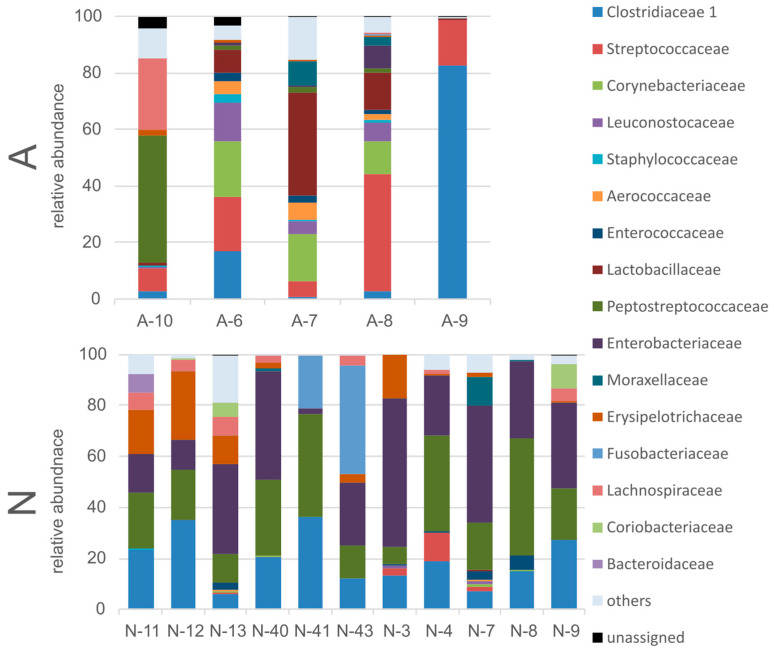
Composition of the polar bear intestinal bacterial community at the family level for individuals within the studied groups.

Most of the annotated bacterial OTUs were assigned to 16 families (Figure 6), although representatives (not from each of these families) were found in both studied groups of bears. The most abundant bacterial families in the intestine microbiome of the N-bear group were Enterobacteriaceae, Peptostreptococaceae, Clostridiaceae, Eryseopelotrichaceae, and Fusobacteriacea (relative abundance >5%). Although, it should be mentioned that not all studied bears from this group harbored representatives of all these families, except the first three. The most abundant bacterial families in the A-group were Streptococaceae, Clostridiaceae, Lactobacillaceae, Corynebacteriaceae, Peptostreptococaceae, and Leuconostocaceae. All the bears in this group harbored representatives of all these families, with the only exception of Corynebacteriaceae. Furthermore, the differences in the relative abundance of representatives of Enterobacteriaceae, Peptostreptococaceae, Clostridiaceae, Streptococaceae, Lactobacillaceae, and Leuconostocaceae were statistically significant (Mann–Whitney U Test, *p* < 0.05).

**Figure 7:**
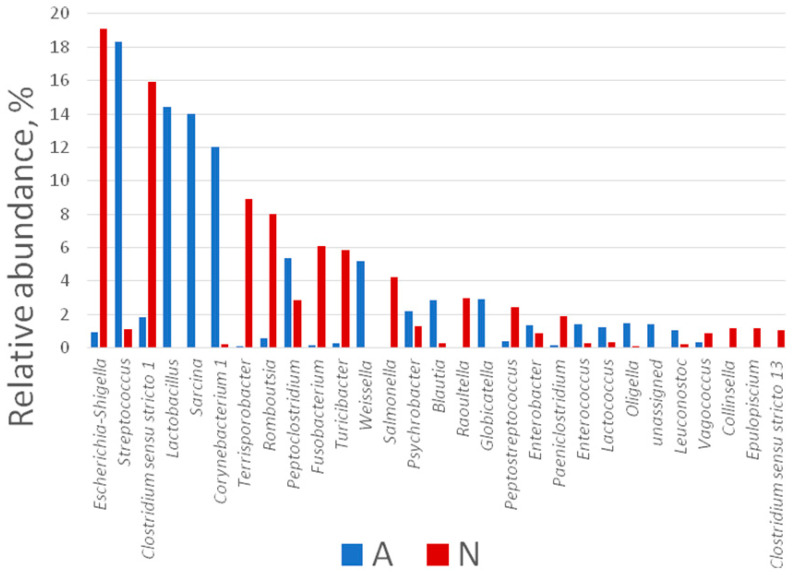
Composition of the polar bear intestinal bacterial community at the genus level.

A total of 121 genera of bacteria were detected in both studied groups of polar bears. It should be noted that we considered only genera that were found in more than one individual and in the amount of more than 0.1%. The most abundant genera in the microbiome of the N-bears were representatives of the *Escherichia*/*Shigella* group, as well as *Clostridium*, *Terrisporobacter*, and *Roumboutsia*, while those for the A-group were *Streptococcus*, *Lactobacillus*, *Sarcina*, and *Corynebacterium* (Figure 7). More than that, the intestine microbiome of A-bears harbored more OTUs attributed to unique genera: six (*Raoutella*, *Peptostreptococcus*, *Epulopiscium*, *Cedecea*, *Bacteroides*, and *Edwardsiella*) and one (*Globicatella*) unique genera for A- and N-bears, respectively (with a relative abundance >0.5%). The differences in the microbiome composition assessed at the genus level were found to be significant (Mann–Whitney U test, *p* < 0.05) for all dominating genera (with a relative abundance >5%), with the exception for *Fusobacterium, Turicibacter,* and *Peptoclostridium*.

### 3.3. Gut Fungal Community of the Polar Bears

**Figure 8:**
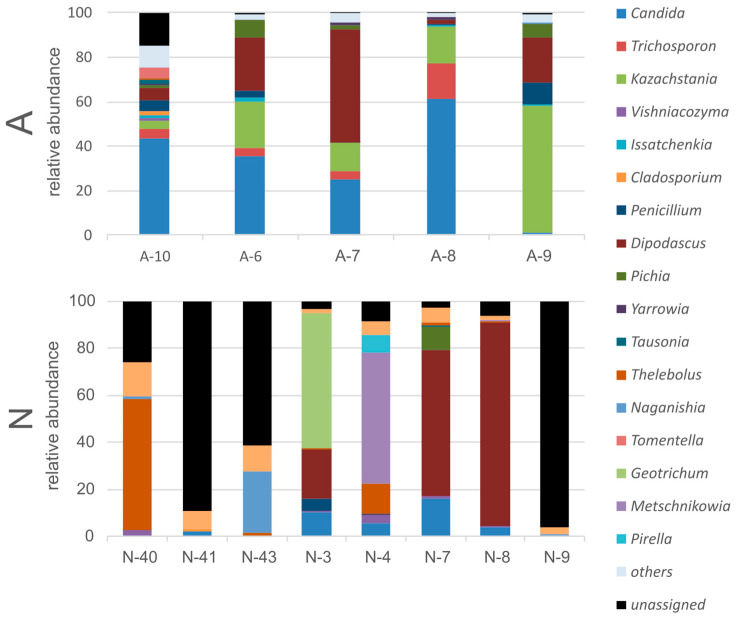
Composition of the polar bear intestinal fungal community at the genus level for individuals within the studied groups.

In total, 1,270,568 reads were obtained for both the N-group and A-group samples. However, it turned out that the proportions of the fungi-annotated reads for the two studied groups differed significantly: 37.9% of the obtained reads for the N-bears were annotated as non-fungal, while in the A-bears, this proportion was only 6.3%. More than that, the A-bear fungal community consisted of 23 genera (detected for more than one individual and with abundance of more than 0.1%), while N-bears consisted only of 16 fungal genera (Figure 8). The dominant fungal genera in N-bears were *Thelebolus* (*globosus*), *Mrakia*, *Dipodascus* (*australiensis*), *Candida* (*sake*), and *Geotrichum*. Representatives of *Thelebolus* (*globosus*) were absolute dominants in more than a half of the studied bears from the N-group. However, we determined representatives of this genus only in one individual of the A-group. The majority of the obtained reads in A-bears were attributed to the genera *Candida* (*tropicalis*), *Kazachstania* (*slooffiae*), and *Trichosporon*.

## 4. Discussion

As the ice-free period in the Arctic increases, it becomes harder for polar bears to survive the summer season on land, relying on fat stores gained in spring. In our sample, there were two groups of bears captured during the end of ice-free season. Polar bears that were feeding at the human waste landfill appeared to be in similar physical conditions to the bears on their natural diet. More than that, feeding on human waste did not cause any signs of intestinal dysbiosis: there are no signs of community disruption, notable decrease in overall microbial diversity, or a critical decrease in the evenness of the community structure. Therefore, access to anthropogenic food source could potentially increase the survival potential of animals. However, the health risks associated with this type of feeding have to be assessed for sound conclusions.

Despite the fact that our sample of polar bears included males and females, as well as individuals of different age groups, it was impossible to identify any patterns between age and sex characteristics and the intestinal microbiome composition of the bears, although we recognized that this disability resulted from the limited number of samples.

The alpha-diversity indexes calculated for the intestinal microbiome of the bears of the Karo–Barents subpopulation were similar to the previously obtained data for other populations of polar bears [16,17]. Despite the absence of statistically significant differences in the studied indexes for both groups of bears, we have noted some shifts in the alpha-diversity indexes associated with changes in their feeding habits. Thus, the A-group was characterized by higher levels of the Chao index, while the Shannon and Simpson indexes were somehow lower than those of the N-group. The Chao index reflects the changes in the total richness of the ecosystem (in our case, intestinal microbial community), supposing slight increase in the diversity of intestinal microorganisms. The intestinal communities of herbivorous animals are characterized by a greater diversity in comparison to carnivores [29].

The composition of the gut bacterial microbiome of both groups of the studied polar bears at the phylum level coincides with previously studied polar bear intestinal communities [16,17]. Moreover, such a composition of the microbiome, in general, is quite typical for various mammalian species [30]. However, a small relative abundance of representatives of the phylum Verrucomicrobia is noteworthy, although these differences can be associated with various methodological features.

However, at a lower taxonomic level, we determined the differences in the intestinal bacterial communities for the two studied groups of bears. Such differences may be related to either nutritional adaptation (shifts in feeding habits) or general stress [31,32]. Nevertheless, very high levels of intra-group variability in the bacterial microbiome composition obstruct the determination of inter-group differences. Perhaps this variability is associated with the irregular feeding of polar bears, which leads to the formation of contrasting conditions in the intestine.

Polar bears on their natural diet have a very similar composition of the gut community to other terrestrial carnivores or omnivores [29,30]. However, we did not recognize any members of the intestinal communities typical for the marine mammals [33]. The occurrence of ‘marine’ Fusobacteria in some individuals is apparently associated with the recent consuming of a seal, a typical host of these microorganisms [34]. To some extent, we also can attribute to the marine-mammal-associated microorganisms *Peptoclostridium* (the obtained sequence is identical to KM100438.1, previously isolated from the feces of the Weddell seal), as well as *Terrisporobacter* and *Paeniclostridium* (whereby identical sequences JQ203739.1 and JQ202995.1, respectively, were previously identified in the feces of bottlenose dolphins, and also livestock and humans). Also, we found no specific features of the microbiome that can be typical for insectivorous animals. Furthermore, the polar bear gut microbial community differs from that of the brown bear [35], which is its closest phylogenetic relative [1].

The composition of the gut community of the polar bears that are feeding on human waste dump resembles the intestinal communities of neither marine mammals, nor predatory mammals in general. The presence of numerous fermentative bacteria as *Lactobacillus*, *Leuconostoc*, and *Weissella,* as well as the reduced proportion of Enterobacteriaceae (*Escherichia*/*Shigella*) make these bears more similar to terrestrial and marine herbivorous mammals [30,33]. Such changes can be explained by a decrease in the proportion of proteins and fats originated from the natural food objects of the polar bear (seals) and an increase in the proportion of complex carbohydrates that are common food waste objects in the dump. Two other bacterial genera that dominate in the A-bears’ intestinal community (*Streptococcus* and *Globicatella*, both from the order Lactobacillales) are not known to have a clear functional role or be associated with any particular type of digestion.

The metabolic profile of the intestinal bacterial community revealed the presence of some enzymes involved in the decomposition of plant polymers and chitin; however, their amount is not enough to speculate about their functional role in the host metabolism. Most likely, polar bears are not able to assimilate plant and invertebrate polymers either by digestion or fermentation. This is also supported by the morphology of their digestive system, which does not include blind appendices and voluminous chambers, as in fermentative marine [33,36] and terrestrial animals [37].

The remarkably low number of the detected fungal sequences in the ITS library of the N-bears can be explained by a lesser amount of the required matrix (fungal DNA). This can be explained by an extremely poor fungal community in the feces of N-bears. The few reads that were annotated as fungi were predominantly represented by the genera *Thelebolus* and *Dipodascus*. Sequences similar to those of *Thelebolus* were found both in the soils and ices of the Antarctic (sequence nos. MT367247.1 and MK889369.1), and in the Canadian Arctic (sequence no. LC514937.1). Sequences similar to *Dipodascus* were found in human feces (KC143379.1) and sputum (OW983049.1), as well as in non-animal environments. Two other dominants, *Candida* (*sake*) and *Geotrichum*, are also eurybiontic organisms not associated with animal loci. The estimated low abundance and low reproducibility of the data obtained for the individuals of the N-group suggest that the fungal community in N-bears is represented by random transit fungi.

The fungal community of the A-group of bears was dominated by *Candida* (*tropicalis*), *Kazachstania* (*slooffiae*), and *Trichosporon*. These fungi are facultative pathogens, well-known in veterinary and medical practice. Moreover, *Trichosporon* was previously known as a wound pathogen of marine mammals [38]. Also, it is important to note that the abundance of non-fungal ITS reads in the bears of the A-group was very low. This means that the feces of these bears are quite densely populated with fungi. Considering the relatively low intra-group variability in the obtained abundance of the annotated reads, it may be assumed that the bears from the A-group harbor a relatively stable fungal community.

Thus, normally, the intestinal tract of polar bears does not contain a significant amount of yeast and is only slightly contaminated with environmental fungi. However, contact with human waste promotes its colonization with facultative pathogens.

### Limitations

We examined all bears feeding on the waste dump during the study period. However, there were only five of them. Therefore, the data obtained should be approximated with caution. Further research is needed to achieve greater certainty.

## 5. Conclusions

Thus, we have shown the changes in the intestinal microbial community of the polar bear resulting from a short-term transition to feeding on untypical and unnatural food objects. The absence of any signs of digestive stress and the presence of signs of adaptive changes in the microbiome along the path of optimizing the assimilation of plant substrates were noted. It is likely that such changes are very rapid and indicate a high variability of the intestinal microbiome. This is also evidenced by the colossal level of inter-individual variability, rarely observed in mammals.

In addition, we have shown for the first time the composition of the fungal community of the polar bear intestine. It was concluded that it strongly depends on the habitat of the animal. In natural environments and on a natural diet, the intestinal community of the polar bears contains practically no fungi. However, proximity to human habitation (and especially, transition to the consumption of human waste objects) leads to the formation of a specific community, with opportunistic pathogenic yeasts of veterinary and medical importance.

## Figures and Tables

**Table 1:** State conditions of the studied polar bears and some measurement of their bodies.

Individual Number	Location	Sex	Age Class	Weight, kg	Body Length, cm	Axillary Girth, cm	Subjective Fatness Index	Body Condition Index (BCI)
N-3	NZ	F	subad	123	166	100	4	−0.56
N-4	NZ	M	cub	41.5	115	70	3	−0.38
N-7	NZ	F	subad	132	180	98	2.5	−1.38
N-8	NZ	F	subad	95	162	87	3	−1.41
A-7	NZ	F	ad	235	194	132	4	0.23
A-9	NZ	F	ad	270 *	187		4	
A-6	NZ	F	ad	245	185	140	4	1.07
A-10	NZ	F	ad	208	190	125	4	−0.05
A-8	NZ	M	ad	562	230	190	4	1.81
N-9	NZ	M	ad	400 *	242		4	
N-40	FJL	F	subad	191	181	118	3	0.24
N-41	FJL	F	ad	299	197	138	4	1.11
N-43	FJL	M	subad	199	180	122	3	0.51
N-11	NZ	M	subad	235	192	125	3	0.36
N-12	NZ	M	ad	247	210	127	2	−0.67
N-13	NZ	F	ad	147	180	147	2	−0.88

Notes: * Calculated mass; NZ, Novaya Zemlya archipelago; FJL, Franz Josef Land archipelago

**Table 2:** Alpha-diversity indexes of the polar bear intestinal community.

Alpha-Diversity Index	A-Bears	N-Bears	Mann–Whitney U Test, *p*-Levels
Chao1	92.30 ± 31.11	59.50 ± 37.35	0.078
Shannon	3.78 ± 1.52	4.24 ± 0.53	0.257
Simpson	0.78 ± 0.23	0.84 ± 0.05	0.486
OTU	95.00 ± 31.91	55.59 ± 28.19	0.497

## Data Availability

All sequencing data are available from the National Center for Biotechnology Information (NCBI) under accession numbers PRJNA910582 (SAMN32133571–SAMN32133596) and PRJNA910225 (SAMN32119049–SAMN32119077).

## Supplementary Materials

The following supporting information can be downloaded at: https://www.mdpi.com/article/10.3390/ani13132067/s1, Table S1: Composition of the polar bear intestinal bacterial community at the OTU level for individuals within the studied groups. NZ, Novaya Zemlya archipelago; FJL, Franz Josef Land archipelago.
